# Study on the Pathway Mechanism of Social Support and Quality of Life Between Self‐Disclosure and Stigma Among Breast Cancer Patients During Recovery

**DOI:** 10.1155/tbj/8826025

**Published:** 2026-08-30

**Authors:** Siyin Lin, Beien Zhang, Shuxian Yu, Chao Wang, Jiaxing Zheng, Zhixin Cao, Hongtao Xu, Guanhua Fan

**Affiliations:** ^1^ Health Management Center, Affiliated Cancer Hospital of Shantou University Medical College, Shantou 515041, Guangdong, China; ^2^ Department of Nursing, Shantou University Medical College, Shantou 515041, Guangdong, China, stu.edu.cn; ^3^ Department of Science and Education, Affiliated Cancer Hospital of Shantou University Medical College, Shantou 515041, Guangdong, China; ^4^ Department of Nursing, Affiliated Cancer Hospital of Shantou University Medical College, Shantou 515041, Guangdong, China; ^5^ School of Public Health, Shantou University, Shantou 515041, Guangdong, China, stu.edu.cn; ^6^ Department of Preventive Medicine, Shantou University Medical College, Shantou 515041, Guangdong, China, stu.edu.cn; ^7^ Center for Nursing Research, Shantou University Medical College, Shantou 515041, Guangdong, China, stu.edu.cn; ^8^ Section of Medical Humanities, Shantou University Medical College, Shantou 515041, Guangdong, China, stu.edu.cn

**Keywords:** breast cancer, quality of life, self-disclosure, social support, stigma, structural equation modeling

## Abstract

**Background:**

Breast cancer comprehensive therapies often cause body image changes, triggering stigma that impairs mental health and quality of life. Existing studies lack an integrated model to reveal the underlying mechanisms between these variables.

**Methods:**

A cross‐sectional survey was conducted among 292 breast cancer patients selected via convenience sampling from 5 hospitals in Guangdong Province between March 2021 and March 2022. The Distress Disclosure Index (DDI), Consumer Experiences of Shame Questionnaire (CESQ), Functional Assessment of Cancer Therapy‐Breast (FACT‐B), and Multidimensional Scale of Perceived Social Support (PSSS) scales were administered. Pearson correlation analysis explored variable relationships, structural equation modeling (SEM) constructed the chain mediating model, the Bootstrap method tested mediating effects, and subgroup associations with stigma were analyzed.

**Results:**

Breast cancer patients had self‐disclosure and stigma scores of 38.17 ± 0.54 and 13.74 ± 0.42, respectively. Stigma was significantly negatively correlated with self‐disclosure, social support, and quality of life (all *p* < 0.01). Self‐disclosure was directly associated with lower stigma (*β* = −0.52, *p* < 0.001) and indirectly mediated by two pathways: the independent pathway through “better quality of life” (11.6% of total effect) and the chain pathway through “higher social support and better quality of life” (5.4% of total effect). Subgroup analysis indicated that patients with a bachelor’s degree or above were more significantly associated with low stigma (OR = 0.42, 95% CI: 0.17–0.96), while introverted patients showed a stronger association with high stigma than extroverted ones (OR = 2.90, 95% CI: 1.57–5.56).

**Conclusion:**

Breast cancer patients exhibit relatively low self‐disclosure and moderate‐to‐low stigma. Higher self‐disclosure is associated with lower stigma directly or indirectly via the chain mediation of social support and quality of life. Enhancing psychological counseling, social support, and quality of life, paired with targeted interventions for education and personality subgroups, facilitates physical and mental recovery and social reintegration.

## 1. Introduction

According to the 2023 Global Burden of Disease Study estimates, breast cancer has become the most commonly diagnosed cancer and the top cause of cancer death among women globally, responsible for 2.3 million incident cases and 764,000 deaths in 2023 alone [1]. Despite advancements in treatment improving survival rates, comprehensive surgeries often alter body image, triggering significant stigma that impairs mental health and quality of life (QOL) [2–6]. Stigma involves internalized shame and social exclusion, hindering help‐seeking and treatment adherence—effects particularly pronounced in sociocultural contexts where illness is taboo [7–10]. Beyond the individual, this burden extends to families as “associative stigma,” deeply rooted in prejudices regarding gender roles [11–13].

In the context of global psycho‐oncology, self‐disclosure extends beyond mere communication; it functions as a vital behavioral strategy for psychological adaptation [14]. Recent global meta‐analyses and cross‐cultural studies indicate that open disclosure in a safe environment helps patients integrate traumatic experiences, whereas silence often leads to harmful rumination on negative emotions [15–17]. This mechanism aligns with social cognitive processing theory and Corrigan’s model of internalized shame: when patients internalize societal stereotypes, they absorb stigma [18, 19]. Active self‐disclosure disrupts this internalization process, serving as a prerequisite for mobilizing social resources and countering stigma [14, 20].

Although social support consistently determines mental health outcomes through interpersonal emotion regulation, fewer than one‐third of studies ground these findings in theoretical frameworks [16, 21]. Social connection theory and the “stress‐buffering model” propose that social support can improve health outcomes and QOL by buffering stress and enhancing self‐efficacy [22–26]. By strengthening psychological resilience and reducing loneliness, social support acts as a critical resource for psychological adaptation and a protective factor against the isolating effects of illness [27–29].

QOL is a multidimensional indicator evaluating the overall health and well‐being of breast cancer patients. Recent high‐quality evidence highlights that QOL is influenced by modifiable determinants; specifically, structured physical activity and exercise have been shown to significantly improve QOL among both breast cancer survivors and the broader cancer population [30, 31]. While stigma negatively impacts QOL, emerging research suggests this relationship is bidirectional. Patients with a higher QOL—characterized by better physical functioning, emotional stability, and social participation—possess greater psychological capital and resilience. This elevated functional state empowers them to resist societal stereotypes and cognitive distortions, thereby actively reducing their perception of internalized stigma [12, 32–37]. Incorporating QOL as an antecedent to stigma reduction is essential for understanding patients’ complete adaptation processes.

In summary, self‐disclosure, social support, QOL, and stigma are closely interrelated and collectively influence the physical and mental outcomes of breast cancer patients. However, existing studies have mostly explored partial relationships in isolation, lacking an integrated model to reveal underlying mechanisms.

Rather than applying these theories in isolation, this study integrates social cognitive processing theory, stress‐buffering theory, and social learning theory to construct a chain mediation model within an integrated “behavior–environment–state–outcome” framework. Guided by social cognitive processing theory, self‐disclosure is conceptualized as the initial proactive behavior. This behavior activates the stress‐buffering model, where social support serves as an environmental resource that mitigates illness impact and enhances the patient’s functional state (QOL). Finally, consistent with social learning theory, the reciprocal interaction between these individual and environmental factors determines the cognitive psychological outcome, thereby reducing the perception of stigma. Specifically, it is hypothesized that self‐disclosure exerts an indirect effect on stigma primarily through the chain path of “social support ⟶ QOL.” Based on the above literature review, the following hypotheses are proposed:

 H1: Self‐disclosure is positively associated with social support.
 H2: Social support is positively associated with QOL.
 H3: QOL is negatively associated with stigma.
 H4: Social support and QOL play a chain mediating role between self‐disclosure and stigma.

**FIGURE 1:**
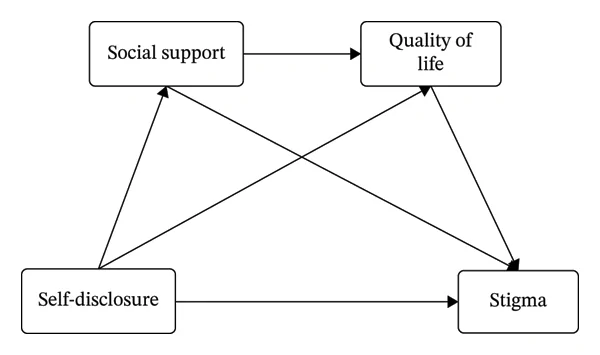
Hypothetical model diagram.

The hypothetical model of the study is illustrated in Figure 1.

## 2. Methods

### 2.1. Study Subjects

This study adopted a cross‐sectional questionnaire design. A total of 292 breast cancer patients admitted to the oncology or breast surgery departments of 5 hospitals in Guangdong Province, China (3 in Shantou, 1 in Guangzhou, and 1 in Shenzhen), from March 2021 to March 2022 were selected as study subjects using convenience sampling.

Eligible patients were recruited face‐to‐face by trained research staff from the oncology and breast surgery units. Recruitment occurred both during inpatient admissions and at scheduled outpatient follow‐up appointments. All potential participants underwent a standardized screening process to verify they met the inclusion criteria and had a clear understanding of the study purpose and procedures. Only after providing either verbal or written informed consent did participants begin the questionnaire.

The inclusion criteria were as follows: (1) pathologically confirmed breast cancer diagnosed according to the 2018 version of the NCCN Clinical Practice Guidelines in Oncology for Breast Cancer Screening and Diagnosis, (2) having undergone major surgical treatments such as mastectomy, breast‐conserving surgery, or breast reconstruction, (3) aged ≥ 18 years, (4) clear consciousness and ability to cooperate with investigators, and (5) informed of and consenting to participate in the study.

The exclusion criteria were as follows: (1) patients with concomitant active malignant tumors other than breast cancer, (2) patients with a history of psychiatric disorders, cognitive dysfunction, or significant sensory impairment (visual or auditory) that would impede their ability to complete the self‐administered questionnaire, and (3) patients with severe acute or chronic physical illnesses (e.g., decompensated cardiopulmonary insufficiency) that would preclude meaningful participation in the study.

Prior to enrollment, all participants provided written informed consent following a comprehensive explanation of the study objectives, risks, and benefits. The study protocol was reviewed and approved by the institutional review board, with Ethics Approval No. SUMC‐2021‐54.

Based on the sample size standards for structural equation modeling (SEM), the final sample of 292 participants exceeds the typical sample size of approximately 200 cases, adheres to the “variable multiple” principle (typically requiring ≥ 8 times the number of variables), and falls within the “safe range” of 250–500 required for stable model analysis, thus meeting the statistical requirements for testing the hypothesized pathways [38–40].

### 2.2. Study Tools

#### 2.2.1. General Information Questionnaire

A self‐developed structured questionnaire was used to gather two categories of baseline information: (1) demographic characteristics: age, education level, marital status, average monthly household income, and self‐rated personality, and (2) clinical characteristics: family history of breast cancer, tumor stage at diagnosis, pathological type, and surgical intervention type.

#### 2.2.2. Distress Disclosure Index (DDI)

The DDI is a widely used self‐report measure of emotional disclosure. It was first developed by Kahn and Hessling [41], and the Chinese version was adapted by domestic scholar Li [42] to measure individuals’ tendency to disclose private information to others. This scale comprises 12 items, each rated on a 5‐point Likert scale from 1 (“Strongly Agree”) to 5 (“Strongly Disagree”). Six items (Items 2, 4, 5, 8, 9, and 10) are reverse scored. The total DDI score is obtained by summing all item responses, with possible scores ranging from 12 to 60. Higher total scores reflect a greater tendency to disclose one’s distress and negative emotions to others. In the present study, the Cronbach’s *α* for the DDI was 0.942, indicating excellent reliability.

#### 2.2.3. Consumer Experiences of Shame Questionnaire (CESQ)

The CESQ was developed through qualitative interviews by Wahl [43] in 1999. Li et al. [44] localized and applied it to Chinese breast cancer patients in 2019, confirming its good reliability and validity. The questionnaire includes 9 items, evaluating patients’ experiences of shame and discrimination due to illness. It uses a 5‐point Likert scale (0 = “Never” to 4 = “Always”), with a total score ranging from 0 to 36. Higher scores indicate stronger subjectively perceived stigma. In this study, the Cronbach’s *α* coefficient of the Chinese version of CESQ was 0.942, indicating high consistency among items in measuring the latent concept of stigma.

#### 2.2.4. Functional Assessment of Cancer Therapy‐Breast (FACT‐B, Version 4.0)

Health‐related QOL was assessed using the Chinese version of the FACT‐B, Version 4.0. This widely used scale was first developed by Cella and colleagues [45] in 1993, and the Chinese translation and validation were completed by Wan et al. [46] in 2003. The FACT‐B scale comprises five domains: four general cancer‐related domains (physical, social/family, emotional, and functional well‐being) derived from the FACT‐G and one breast cancer‐specific concerns domain. In total, the scale contains 36 items, each scored on a 5‐point Likert scale from 0 (“Not at All”) to 4 (“Very Much”). The overall FACT‐B score is calculated by summing the scores of all items, with possible scores ranging from 0 to 144. Higher scores correspond to better QOL. A key strength of this scale is that it not only measures physical symptoms but also addresses the emotional and social challenges faced by breast cancer patients. In our sample, the FACT‐B demonstrated excellent internal consistency, with a Cronbach’s *α* of 0.921.

#### 2.2.5. Perceived Social Support Scale (PSSS)

The PSSS was developed by Blumenthal et al. [47] in 1987 and revised by Jiang Qianjin [48] to assess the level of subjectively perceived social support in breast cancer patients. It includes 3 support dimensions (“family,” “friends,” and “others”) with 12 items in total, scored on a 7‐point Likert scale (1 = “Extremely Disagree” to 7 = “Extremely Agree”). The total score ranges from 12 to 84. In this study, the Cronbach’s *α* coefficient of PSSS was 0.903, and the *α* coefficients of each dimension ranged from 0.903 to 0.945.

### 2.3. Data Collection Methods

Data were collected via questionnaire surveys, distributed and retrieved on‐site by trained research assistants using standardized instructions. To minimize potential response bias (e.g., social desirability bias) given the sensitive nature of stigma and self‐disclosure, participants were guaranteed anonymity. During the survey, the assistants provided unified guidelines to help participants understand the items without leading their responses. Upon completion, the questionnaires were retrieved and checked on‐site by the researchers to ensure completeness; any missing items were clarified with the participant immediately to maximize the valid response rate. All data entry and cleaning procedures were conducted by two independent researchers using double data entry to verify data accuracy. For missing values, we applied mean imputation for items with ≤ 5% missing responses, while questionnaires with more than 5% missing data were excluded from the final analysis.

**FIGURE 2:**
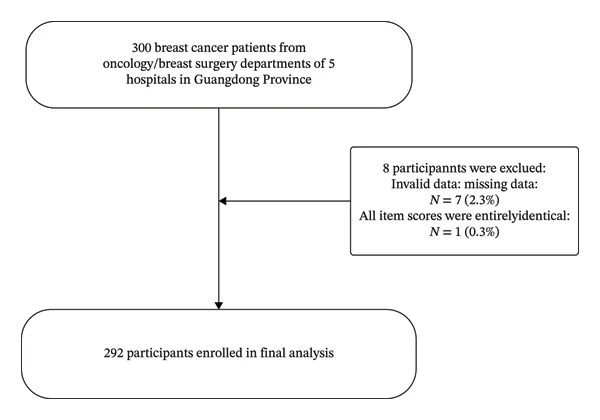
A flowchart about data collection.

A total of 300 questionnaires were distributed to eligible participants. After excluding 8 invalid questionnaires, 292 valid questionnaires were retained for statistical analysis, yielding a valid response rate of 97.3%. A flowchart outlining the complete data collection process is provided in Figure 2.

### 2.4. Statistical Methods

Statistical processing and data analysis were carried out using three software packages: SPSS Version 27.0, AMOS Version 26.0, and R Version 4.4.2. We first used descriptive statistics to characterize the study population. Pearson product–moment correlation analysis was then conducted to investigate the preliminary linear relationships between self‐disclosure, social support, QOL, and stigma. Harman’s single‐factor test was performed to rule out significant common method bias in the dataset. For the SEM analysis, we followed the standard two‐step procedure: First, the measurement model was validated via confirmatory factor analysis (CFA) to ensure data reliability and validity before testing structural paths. Second, the structural model was constructed to test the chain mediating effect of “self‐disclosure ⟶ social support ⟶ QOL ⟶ stigma.” The Bootstrap method (5000 resamples) was used to calculate 95% confidence intervals (CIs) for testing the significance of mediating effects, and model fit was evaluated against established criteria. Additionally, multicollinearity diagnostics were performed before SEM analysis, and subgroup analyses were conducted to explore the associations between population characteristics and stigma. The significance level was set at *α* = 0.05.

#### 2.4.1. Handling of Covariates in the Structural Equation Model

We adopted a theory‐driven approach to construct the serial mediation model, which is the standard practice in psychosocial research examining mediating pathways. Specifically, self‐disclosure was set as the independent variable, stigma as the dependent variable, and social support and QOL as the two consecutive mediating variables.

To maintain model parsimony and enhance the interpretability of the core pathways, demographic and clinical characteristics were not controlled for in the final structural equation model. This decision was made based on three well‐justified methodological and theoretical considerations:

1. Theoretical Rationale: The model aimed to test the core pathways proposed by social cognitive processing theory and the stress‐buffering model—specifically, the mechanism by which self‐disclosure is linked to stigma through social support and QOL.
2. Model Robustness: Given the sample size (*n* = 292), a parsimonious model helps ensure stable parameter estimates. Including multiple low‐prevalence covariates could lead to model overfitting or convergence difficulties.
3. Empirical Justification: Preliminary correlation analyses (Table 1) and group comparisons (Table 2) indicated that most demographic and clinical variables—except for personality and surgery type—showed no significant association with stigma. This supports the decision to focus on the hypothesized psychological pathways.

**TABLE 1:** Correlation analysis between variables of self‐disclosure, stigma, quality of life and social support.

	Self‐disclosure	Stigma	Quality of life	Social support
Self‐disclosure	1			
Stigma	−0.627^∗∗^	1		
Quality of life	0.528^∗∗^	−0.477^∗∗^	1	
Social support	0.694^∗∗^	−0.508^∗∗^	0.491^∗∗^	1

^∗∗^ *p* < 0.01.

**TABLE 2:** Demographic characteristics.

Characteristics	Overall	Low stigma	High stigma	*p* value
Age, *n* (%)				0.239
18–45	93 (31.8)	49 (36.3)	44 (28.0)	
46–69	190 (65.1)	81 (60.0)	109 (69.4)	
> 69	9 (3.1)	5 (3.7)	4 (2.5)	
Education level, *n* (%)				0.120
No education	53 (18.2)	23 (17.0)	30 (19.1)	
Senior high school and below	213 (72.9)	95 (70.4)	118 (75.2)	
Bachelor’s degree and above	26 (8.9)	17 (12.6)	9 (5.7)	
Marital status, *n* (%)				0.775
Unmarried	6 (2.1)	4 (3.0)	2 (1.3)	
Married	267 (91.4)	122 (90.4)	145 (92.4)	
Divorced	9 (3.1)	4 (3.0)	5 (3.2)	
Widowed	10 (3.4)	5 (3.7)	5 (3.2)	
Self‐rated personality, *n* (%)				< 0.001
Introverted	60 (20.5)	16 (10.7)	44 (31.0)	
Ambiverted	119 (40.8)	59 (39.3)	60 (42.3)	
Extroverted	113 (38.7)	75 (50.0)	38 (26.8)	
Average monthly household income per person, *n* (%)				0.582
Income insufficient for expenses	73 (25.0)	30 (22.2)	43 (27.4)	
Balanced income and expenses	136 (46.6)	66 (48.9)	70 (44.6)	
Slight surplus	83 (28.4)	39 (28.9)	44 (28.0)	
Family history of breast cancer, *n* (%)				0.391
None	280 (95.9)	128 (94.8)	152 (96.8)	
Yes	12 (4.1)	7 (5.2)	5 (3.2)	
Chemotherapy, *n* (%)				0.564
None	86 (29.5)	42 (31.1)	44 (28.0)	
Yes	206 (70.5)	93 (68.9)	113 (72.0)	
Time since surgery (months), *n* (%)				0.347
< 1	88 (30.1)	35 (25.9)	53 (33.8)	
1‐2	61 (20.9)	30 (22.2)	31 (19.7)	
> 2	143 (49.0)	70 (51.9)	73 (46.5)	
Breast cancer stage, *n* (%)				0.315
Stage 0	18 (6.2)	12 (8.9)	6 (3.8)	
Stage I	59 (20.2)	26 (19.3)	33 (21.0)	
Stage II	140 (47.9)	66 (48.9)	74 (47.1)	
Stage III	66 (22.6)	26 (19.3)	40 (25.5)	
Stage IV	9 (3.1)	5 (3.7)	4 (2.5)	
Pathological type, *n* (%)				0.687
Noninvasive carcinoma	282 (96.6)	131 (97.0)	151 (96.2)	
Invasive carcinoma	10 (3.4)	4 (3.0)	6 (3.8)	
Surgical type, *n* (%)				0.046
Breast‐conserving surgery	183 (62.7)	75 (55.6)	108 (68.8)	
Mastectomy	97 (33.2)	52 (38.5)	45 (28.7)	
Breast reconstruction surgery	12 (4.1)	8 (5.9)	4 (2.5)	

#### 2.4.2. Handling of Covariates in Subgroup Analyses

**FIGURE 3:**
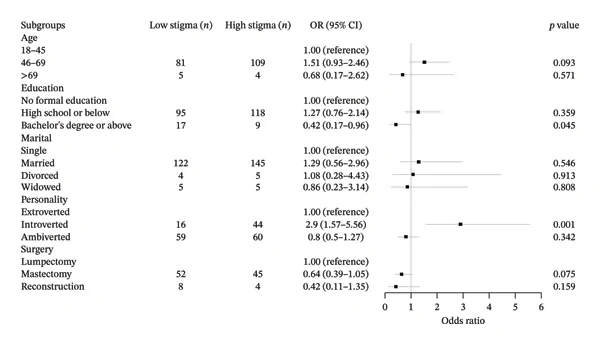
Differences in the association between high and low stigma across different subgroups.

To explore the associations between patient characteristics and stigma, we first dichotomized the total stigma score (CESQ) at the median into low and high groups. Univariate binary logistic regression models were subsequently fitted, where each individual characteristic served as the predictor variable and high stigma was the binary outcome variable. These analyses generated unadjusted odds ratios and 95% CIs, which are presented graphically in Figure 3 (forest plot). This analysis was exploratory, and no adjustment for potential confounders was made due to sample size limitations in specific categories. These preliminary findings identify characteristics potentially associated with high stigma (e.g., introverted personality) that warrant further validation in future longitudinal studies.

#### 2.4.3. Measurement Model Validation and Multicollinearity Diagnostics

To guarantee the robustness of our SEM results, we adopted the widely accepted two‐step analytical procedure. In the first step, we validated the measurement model using CFA. CFA was implemented in AMOS 26.0 software with maximum likelihood estimation to examine the fit of the measurement model prior to testing the hypothesized structural relationships. Convergent validity was established when all standardized factor loadings exceeded 0.50, composite reliability (CR) values were greater than 0.70, and average variance extracted (AVE) values were above 0.50. In the second step, we conducted multicollinearity diagnostics to protect against unstable parameter estimates. The variance inflation factor (VIF) was computed for each independent variable, and values less than 5 were deemed acceptable, indicating no serious multicollinearity problems.

#### 2.4.4. Structural Model and Chain Mediation Analysis

SEM was applied to test the hypothesized chain mediating effect. A path model of “self‐disclosure ⟶ social support ⟶ QOL ⟶ stigma” was constructed. The Bootstrap method (5000 resamples) was used to calculate the 95% CIs for testing the significance of direct and indirect mediating effects.

## 3. Results

### 3.1. Basic Characteristics of the Study Subjects

As shown in Table 2, a total of 292 female breast cancer patients were included in this study. The sample was mainly composed of middle‐aged patients, married patients, and those with an education level of senior high school or below.

An intergroup comparison revealed that the level of stigma was significantly correlated with patients’ personality and surgical type (*p* < 0.05): the proportion of patients with introverted personality in the high‐stigma group (34.4%) was significantly higher than that in the low‐stigma group (12.3%), and the proportion of patients who underwent breast‐conserving surgery was also higher in the high‐stigma group (68.8%). No statistically significant differences were observed in other demographic characteristics.

### 3.2. Common Method Bias Test and Measurement Model Validation

#### 3.2.1. Common Method Bias Test

Harman’s single‐factor test was employed to conduct an exploratory factor analysis on all 69 items spanning four study variables: self‐disclosure, stigma, social support, and QOL. Analysis revealed that the variance explained by the largest common factor was 30.37% (< 40%), suggesting that no significant common method bias was present in the dataset [49].

#### 3.2.2. Measurement Model Validation (CFA)

CFA was conducted to examine the measurement model, including two multidimensional constructs (PSSS, FACT‐B) and two unidimensional constructs (DDI, CESQ). The model showed acceptable fit to the data: *χ* ^2^/d*f* = 3.233, RMSEA = 0.088, CFI = 0.868, TLI = 0.856, and IFI = 0.869. A ratio of normed chi‐square (*χ* ^2^/d*f*) as high as 5 has also been recommended by Wheaton et al. [50], and our observed value of 3.233 fell well within this acceptable range. While an RMSEA of 0.08–0.10 represents mediocre fit [51, 52], and GFI, AGFI, CFI, and TLI fell slightly below the ideal thresholds, all fit indices exceeded 0.8, a level widely considered acceptable in relevant research [53]. Therefore, the proposed model demonstrated adequate fit with the empirical data.

**FIGURE 4:**
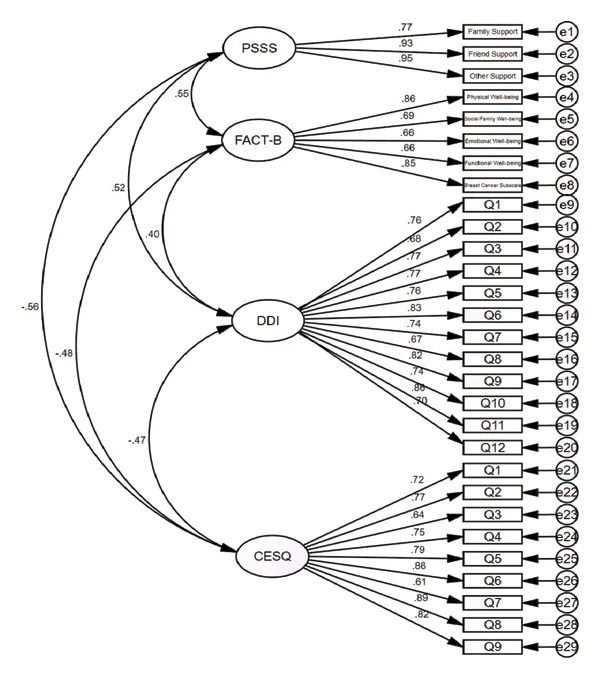
Standardized loadings from confirmatory factor analysis.

As summarized in Table 3, all standardized factor loadings were statistically significant (*p* < 0.001) and exceeded the 0.50 threshold. The CR for all constructs ranged from 0.86 to 0.94, indicating good internal consistency. Moreover, the AVE values were all above 0.50, confirming strong convergent validity. The visualized measurement model with standardized coefficients is presented in Figure 4.

**TABLE 3:** Measurement model validation.

Construct	Indicators	Standardized loading	CR	AVE
Social support (PSSS)	3 dimensions	0.77–0.95	0.92	0.79
Quality of life (FACT‐B)	5 dimensions	0.66–0.86	0.86	0.56
Self‐disclosure (DDI)	12 items	0.67–0.83	0.94	0.58
Stigma (CESQ)	9 items	0.61–0.89	0.93	0.59

### 3.3. Scores of the DDI, CESQ, FACT‐B, and PSSS Among Breast Cancer Patients

Table 4 shows the scores of the DDI, CESQ, FACT‐B, and PSSS in breast cancer patients.

**TABLE 4:** Scores of DDI, CESQ, FACT‐B, and PSSS in breast cancer patients (*x̅* ± *s*, *n* = 292).

Items	Score
Total score of DDI	38.17 ± 0.54
Total score of CESQ	13.74 ± 0.42
Total score of FACT‐B	89.72 ± 1.10
Total score of PSSS	57.88 ± 0.61

### 3.4. Correlation Analysis and Multicollinearity Diagnostics

#### 3.4.1. Pearson Correlation Analysis

We calculated Pearson correlation coefficients to examine the pairwise relationships between the core variables. As presented in Table 1, all bivariate correlations reached statistical significance at the *p* < 0.01 level. Specifically, higher levels of self‐disclosure corresponded to greater social support (*r* = 0.694) and better QOL (*r* = 0.528) and were associated with lower levels of stigma (*r* = −0.627). Social support was positively correlated with QOL (*r* = 0.491) and negatively associated with stigma (*r* = −0.508). Finally, QOL showed a significant inverse correlation with stigma (*r* = −0.477).

#### 3.4.2. Multicollinearity Check

To safeguard the stability of the structural equation model, multicollinearity diagnostics were conducted using multiple linear regression in SPSS. VIF values of 2.014, 1.447, and 2.117 were obtained for social support, QOL, and self‐disclosure, respectively, with corresponding tolerance values ranging from 0.472 to 0.691. All VIF values were well below the conservative threshold of 5, and all tolerance values exceeded the critical cutoff of 0.2, confirming that multicollinearity does not pose a threat to the validity of parameter estimates in the subsequent path analysis.

### 3.5. Chain Mediating Effect of QOL and Social Support Between Self‐Disclosure and Stigma in Breast Cancer Patients

Based on the above correlations, we used a structural equation model to test the mediating effect of self‐disclosure on stigma. In the model setting, self‐disclosure (total score of DDI) was taken as the independent variable, stigma (total score of CESQ) as the dependent variable, and social support (total score of PSSS) and QOL (total score of FACT‐B) as mediating variables, and it was assumed that social support and QOL form a sequential mediating chain.

#### 3.5.1. Model Fit

The results (see Table 5) indicated that all fit indices met the established criteria for model acceptance. Specifically, according to conventional guidelines [54, 55], RMSEA < 0.05 corresponds to good model fit, 0.05 ≤ RMSEA < 0.08 indicates acceptable fit, 0.08 ≤ RMSEA < 0.10 suggests mediocre fit, and RMSEA ≥ 0.10 indicates poor fit. Although the RMSEA value of 0.07 exceeded the strict threshold for “good fit,” it fell within the 0.05–0.08 range, which is characterized as an “adequate” or “reasonable” fit. When considered alongside the other descriptive fit indices (GFI, CFI, and TLI, all > 0.90), these findings suggest that the proposed chain mediating model represents an adequate representation of the observed data.

**TABLE 5:** Test results of model fit.

Fit index	Fit criteria	Fit index	Goodness of fit
*χ* ^2^/d*f*	1∼5	2.30	Fit
RMSEA	< 0.1	0.07	Adequate fit
GFI	> 0.80	0.99	Fit
AGFI	> 0.80	0.96	Fit
CFI	> 0.80	0.99	Fit
IFI	> 0.80	0.99	Fit
NFI	> 0.80	0.99	Fit
TLI	> 0.80	0.98	Fit

#### 3.5.2. Estimation of Path Coefficients

In the structural equation model, the standardized regression coefficients of all main paths reached a significant level (*p* < 0.001), verifying the directional relationships we hypothesized (see Table 6):

 H1 (Self‐disclosure ⟶ Social support): The regression coefficient was 0.694. A higher level of self‐disclosure was correlated with a higher level of social support (*β* = 0.694) standard deviations. There was a significant positive correlation between the two, and there remained an independent promoting effect after controlling for other factors, which was consistent with the correlation analysis.
 H2 (Social support ⟶ QOL): The regression coefficient was 0.241. A higher level of social support was positively associated with better QOL (*β* = 0.241) standard deviations. It remained significant after controlling for factors such as self‐disclosure, confirming the promoting effect of social support on QOL.
 H3 (QOL ⟶ Stigma): The regression coefficient was −0.203. Higher QOL was correlated with lower levels of stigma (*β* = −0.203) standard deviations. The negative relationship was significant, supporting the inference of the mediating role of QOL.

**TABLE 6:** Regression analysis of the chain mediation model of quality of life and social support.

Path	Regression coefficient	*p*
Social support ← Self‐disclosure	0.694	< 0.001
Quality of life ← Self‐disclosure	0.360	< 0.001
Quality of life ← Social support	0.241	< 0.001
Stigma ← Quality of life	−0.203	< 0.001
Stigma ← Self‐disclosure	−0.52	< 0.001

#### 3.5.3. Test of Mediating Effects

Mediating analysis with 5000 Bootstrap resamplings showed that the total effect, direct effect, and indirect effect of self‐disclosure on stigma were all significant (see Table 7).

1. The total effect coefficient was −0.48 (95% CI: −0.55 to −0.42), indicating that for each increase of one standard unit in self‐disclosure, stigma would decrease by 0.48 standard units.
2. The direct effect coefficient was −0.40 (95% CI: −0.48 to −0.33), accounting for **83%** of the total effect, suggesting that the impact of self‐disclosure on stigma is mainly direct.
3. The total indirect effect coefficient was −0.08 (95% CI: −0.13 to −0.04), accounting for **17%** of the total effect.
4. Specific mediating path effects Path 1 (Self‐disclosure ⟶ QOL ⟶ Stigma): The effect value was −**0.06** (95% CI: −0.10 to −0.03), accounting for **11.6%** of the total effect. This single mediating path was significantly established, indicating that part of the role of self‐disclosure is to reduce stigma by improving QOL.

**TABLE 7:** Analysis of the chain mediating effect of quality of life and social support.

Mediating model	Effect value	Effect ratio (%)	95% CI
Total effect	−0.48		[−0.55, −0.42]
Direct effect	−0.4	83	[−0.48, −0.33]
Total indirect effect	−0.08	17	[−0.13, −0.04]
Self‐disclosure ⟶ Quality of life ⟶ Stigma	−0.06	11.6	[−0.10, −0.03]
Self‐disclosure ⟶ Social support ⟶ Quality of life ⟶ Stigma	−0.03	5.4	[−0.05, −0.01]

Path 2 (Self‐disclosure ⟶ Social support ⟶ QOL ⟶ Stigma): The effect value was −**0.03** (95% CI: −0.05 to −0.01), accounting for **5.4%** of the total effect. This chain mediating path also reached a significant level, illustrating that self‐disclosure indirectly reduces stigma by enhancing social support and QOL.

**FIGURE 5:**
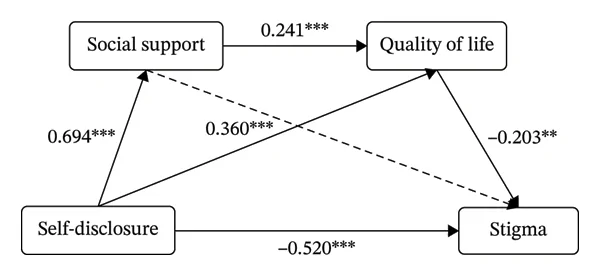
Chain mediating model of social support and quality of life between self‐disclosure and stigma in patients after breast cancer surgery. Note: The dashed line indicates that the path coefficient is not significant.

Both mediating paths were significantly nonzero, indicating that the improvement of patients’ own psychological well‐being and the strengthening of social support networks both play a partial mediating role in reducing stigma (Figure 5).

In summary, the model analysis supports the main hypothesis of this study: Social support and QOL of breast cancer patients play a significant chain mediating role between self‐disclosure and stigma.

### 3.6. Subgroup Analysis

To further explore whether stigma is influenced by different population characteristics, this study conducted a subgroup stratification analysis. According to the results of the subgroup analysis on stigma (Figure 3), subgroups such as age, marital status, and surgical type showed no statistically significant association with stigma. However, individuals with a bachelor’s degree or above had significantly lower stigma than those without formal education (OR = 0.42, 95% CI [0.17–0.96], *p* = 0.045); introverted patients had significantly higher stigma than extroverted patients (OR = 2.90, 95% CI [1.57–5.56], *p* = 0.001).

## 4. Discussion

This study confirms that social support and QOL play a chain mediating role between self‐disclosure and stigma in breast cancer patients. The following discussion will explore the mechanism of this role in combination with the theoretical framework, compare it with existing studies, and explain its implications for clinical nursing practice.

### 4.1. Levels of Self‐Disclosure, Stigma, Social Support, and QOL in Breast Cancer Patients

The survey results of 292 patients showed the following scale scores for the four main variables: self‐disclosure, stigma, QOL, and social support:

The total score of self‐disclosure (DDI scale) in breast cancer patients was **38.17** ± 0.54, which fell within the moderate range (30–44 points). This score is consistent with other studies conducted in China. For example, Wang et al. [56] found that the average DDI score of breast cancer patients during treatment was approximately 37 points, suggesting that the willingness of Chinese breast cancer patients to self‐disclose during treatment is moderate. This may be related to the Chinese cultural norm of reporting good news but concealing bad news and the restrictions on the expression of negative emotions due to the family and social roles of middle‐aged women.

The total score of stigma (CESQ questionnaire) in breast cancer patients was **13.74** ± 0.42, at a moderately low level. This indicates that most breast cancer patients experience mild to moderate stigma in practice. The sample of this study included patients of all ages, and the overall score was slightly lower, which may imply that the issue of stigma has improved in recent years with the enhancement of public awareness of breast cancer and the development of patient education.

The QOL of breast cancer patients was above the moderate level, with a total FACT‐B score of **89.72** ± 1.10. Among the dimensions, the social/family and functional dimensions recovered relatively well, but the emotional dimension and breast‐related concerns were the main troubles. This may be because physiological functions recover after treatment and family support is effective, but persistent psychological concerns about recurrence and body image restrict further improvement in QOL. In addition, QOL can change dynamically with treatment stages [33], and this temporal characteristic requires in‐depth exploration in subsequent studies.

The overall social support of breast cancer patients was above the moderate level, with a total PSSS score of **57.88** ± 0.61. The sources of support were most prominent from family, followed by friends, and limited from other social relationships, which is consistent with the core role of families in disease care in Eastern culture. It is suggested that while consolidating family support, efforts should be made to expand patients’ peer support networks to improve their psychological adaptation.

### 4.2. Analysis of Mediating Effects

#### 4.2.1. Mediating Role of QOL Between Self‐Disclosure and Stigma in Breast Cancer Patients

Mediating analysis showed that QOL played a partial mediating role between self‐disclosure and stigma (with the indirect effect accounting for 11.6%). From the perspective of social cognitive processing theory, self‐disclosure promotes the emotional processing and cognitive integration of patients’ disease experiences, improves QOL, and enhances self‐worth, thereby weakening the foundation of stigma. The results of this study are consistent with existing literature: A systematic review by Mokhtari‐Hesari and Montazeri confirmed that psychological interventions can significantly improve the QOL of breast cancer patients [38, 57]; a study by Yeung et al. found that self‐stigma reduces QOL through the mechanism of sense of burden [58]; this study, on the other hand, conversely demonstrated the buffering effect of QOL on stigma, improving the “self‐stigma–psychological medium–outcome” theoretical model proposed by Corrigan and Watson [18].

Bootstrap analysis showed that the effect value of this path was −0.06 and significant, indicating that self‐disclosure can reduce stigma by improving QOL without relying on social support, highlighting the independent value of QOL in clinical interventions. This pathway is further supported by intervention‐based research; recent systematic reviews and meta‐analyses have demonstrated that structured physical activity and exercise interventions significantly enhance health‐related QOL among breast cancer survivors and cancer patients, highlighting QOL as a modifiable target for psychosocial interventions [30, 31]. Based on this, it is suggested that medical staff should improve patients’ QOL through symptom management, psychological counseling, and maintenance of social functions, so as to improve their self‐evaluation and reduce stigma. This is highly consistent with the concept proposed by Holzner et al. that QOL should be regarded as a core indicator in oncology nursing [58].

#### 4.2.2. Chain Mediating Role of Social Support and QOL Between Self‐Disclosure and Stigma in Breast Cancer Patients

This study revealed that self‐disclosure is linked to stigma through the chain path of “social support ⟶ QOL,” with the mediating effect accounting for 5.4%, clarifying the interaction logic of variables.

This empirical path confirms the integration of our theoretical framework, demonstrating how behavioral disclosure facilitates environment‐based resources to optimize the internal state and cognitive outcomes.

The study found that self‐disclosure was highly positively correlated with social support (*r* = 0.694, *β* = 0.694), which is a key prerequisite for patients to obtain social support, consistent with the research conclusion of Taniguchi et al. Self‐disclosure provides a basis for social support and enhances its positive effects and is called a “booster” of supportive effects [54, 55, 59]. Therefore, when encouraging patients to disclose, it is necessary to simultaneously build a supportive environment to avoid negative feedback after disclosure aggravating stigma, highlighting the importance of clinically balancing “cultivation of disclosure ability” and “construction of support networks.”

The model showed that social support had a direct promoting effect on QOL (*β* = 0.241): from the perspective of stress‐buffering theory, social support alleviates physical and mental stress through emotional comfort, practical help, and effective information, thereby improving QOL; a longitudinal study by Rüsch also confirmed that individuals who actively seek support can establish a more stable support network and achieve better long‐term adaptation effects [60–62]. The two together form a dual “buffer” against stigma, forming a positive cycle: disclosure leads to support, and support improves QOL, which in turn enhances patients’ self‐esteem and sense of hope, promoting their transformation from “disease victims” to “active survivors” and ultimately resolving stigma.

This suggests that interventions need to adopt a “multilevel linkage” strategy, and the lack of any link will limit the effect. Guide disclosure to activate the support system, rely on support resources to improve QOL, and ultimately alleviate stigma.

In summary, the chain mediating model deepens the understanding of the psychosocial adjustment process of breast cancer patients: self‐disclosure (individual behavior) and social support (external environment) jointly affect QOL (health status), which in turn determines stigma (psychological outcome). This is consistent with the stress–social support–outcome framework [25] and shifts the perspective from “psychological distress–QOL” to “disclosure–stigma,” both emphasizing the multiplicity of mediating mechanisms.

### 4.3. Differences in the Association Between High and Low Stigma Across Different Subgroups

The subgroup analysis revealed a potential association between the educational level and stigma: individuals with a bachelor’s degree or above showed lower stigma compared to those without formal education (OR = 0.42, 95% CI: 0.17–0.96, *p* = 0.045). This pattern aligns with the “educational buffering hypothesis,” which posits that education may mitigate psychological distress by enhancing coping resources [9]. However, given the highly imbalanced distribution—only 26 participants held a bachelor’s degree or above—this finding should be interpreted with caution. The small sample size in higher education categories limits the robustness and generalizability of this result, and further research with more balanced educational samples is warranted.

Personality traits also appeared to influence stigma perception, with introverted patients showing a stronger association with high stigma than extroverted patients (OR = 2.90, 95% CI: 1.57–5.56, *p* = 0.001). This may be partly explained by the lower tendency of introverted individuals to engage in self‐disclosure, which was found in this study to be linked to stigma through the chain mediation of social support and QOL. Nevertheless, these subgroup findings are exploratory and should be confirmed in future studies with larger and more diverse samples. In summary, while both educational level and personality traits may contribute to stigma among breast cancer patients, the results concerning education are constrained by sample limitations. Personality, particularly through its influence on disclosure behavior, appears to play a more robust role in shaping stigma perception.

## 5. Practical Implications for Psycho‐oncology and Public Health

Our chain mediation model moves beyond simple associations to provide a “multilevel linkage” framework for clinical action. To improve the social reintegration of breast cancer survivors, we propose the following strategies:

Clinical Intervention Models: Hospitals should transition from a survival‐centric care model to a “reintegration‐centric” one. We recommend a “behavior–environment–state” intervention model: first, provide “safe disclosure” training for patients to facilitate proactive behavior; second, implement family‐centered counseling to optimize the social buffer; and finally, use symptom management to stabilize the patient’s functional state (QOL).

Targeted Patient Support: Support strategies must be differentiated. Introverted patients and those with lower education levels are high‐risk subgroups for stigma. For these populations, we suggest peer‐led mentor programs where long‐term survivors provide modeled disclosure behaviors [63], thereby bridging the gap that formal clinical care might miss.

Clinical Screening Programs: Systematic psychosocial screening should be integrated into routine postoperative follow‐ups. Standardized tools such as the CESQ and DDI should be used as “psychological vital signs” to identify patients at risk of internalized shame before it impairs their treatment adherence.

Policy Implications: Health authorities should include psychosocial recovery metrics—such as stigma levels and social support scores—into the quality‐of‐care audit standards for oncology centers. This would incentivize hospitals to invest in professional psychological counseling resources alongside surgical excellence.

## 6. Limitations

This study advances our understanding of the relationship between self‐disclosure and breast cancer stigma, but several methodological limitations should be noted.

First, the cross‐sectional design of this study prevents us from drawing unambiguous causal inferences. Although SEM allows us to test theoretically derived pathways, it cannot confirm the temporal ordering of variables. Future longitudinal studies that follow breast cancer patients over time are essential to verify the directionality of the observed associations and establish definitive causal relationships. Second, the representativeness of our findings is constrained by the sampling strategy. We utilized convenience sampling from five hospitals exclusively within Guangdong Province. As a highly developed coastal region with distinct socio‐economic and cultural characteristics (such as the Cantonese “reporting good news but not bad” culture), the experiences of our participants may not fully mirror those of patients in inland or rural areas of China. This potential regional bias and the nonrandom sampling approach limit the external validity of our results. Third, our control for potential confounding variables was insufficient. Notably, we did not collect data on whether patients had received psychological counseling, which could significantly influence both stigma and QOL. Finally, the reliance on self‐reported scales may introduce social desirability bias, as patients might under‐report sensitive feelings such as stigma. Future studies should adopt a multicenter approach with randomized sampling, incorporate important confounders such as psychological counseling, and integrate qualitative methods to capture more nuanced psychological experiences.

## 7. Conclusion

This study explored the relationships between self‐disclosure, stigma, social support, and QOL among breast cancer patients, drawing important conclusions: The overall stigma level of breast cancer patients was moderately low, and stigma was more pronounced among patients with a low educational level and introverted personality. Self‐disclosure, social support, and QOL all scored above the moderate level and were significantly correlated with stigma. Patients with a higher degree of self‐disclosure have more social support, better QOL, and lower stigma, which verifies the applicability of the social cognitive processing theory and the stress‐buffering model in this population; social support and QOL play a chain mediating role between self‐disclosure and stigma, which verifies the systematic integration of the social cognitive processing theory and the stress‐buffering model within the broader context of social learning theory. Self‐disclosure is indirectly associated with lower levels of stigma either through the single pathway of improving QOL or through the sequential pathway of “social support ⟶ QOL.” In addition, the educational level has a protective effect on stigma, with individuals with higher education showing significantly lower stigma, while introverted personality is associated with higher stigma. This suggests that intervention measures need to consider individuals’ educational backgrounds and personality traits and adopt differentiated strategies.

## Author Contributions

Siyin Lin: writing–review and editing, writing–original draft, visualization, supervision, software, resources, project administration, methodology, investigation, formal analysis, data curation, and conceptualization. Beien Zhang: writing–review and editing, writing–original draft, resources, project administration, methodology, data curation, and conceptualization. Shuxian Yu: writing–review and editing, methodology, and conceptualization. Chao Wang: writing–review and editing, methodology, and conceptualization. Jiaxing Zheng: writing–review and editing, methodology, and conceptualization. Zhixin Cao: writing–review and editing, methodology, and conceptualization. Hongtao Xu: writing–review and editing, methodology, and conceptualization. Guanhua Fan: writing–review and editing, writing–original draft, methodology, data curation, conceptualization, and project administration.

## Funding

This work was supported by the General Program of Guangdong Natural Science Foundation (Grant Nos. 2022A1515012192 and 2025A1515010750), the Medical Research Fund Project of Guangdong Province (Grant Nos. A2022535 and B2026304), the 2025 Nursing Discipline Research Project of Chinese Medical Association Publishing House (Grant No. CMAPH‐NRD2025037), the 2025 Annual Research Project on Undergraduate Teaching Reform of Clinical Teaching Bases in Guangdong Province (Grant No. 2025JD014), and the Science and Technology Plan Project of Shantou City (Medical and Health Category), Guangdong Province, China (Project No. 250804096497979).

## Disclosure

All authors have read and approved the final version of the manuscript.

## Ethics Statement

The study was approved by the Ethics Committee of Shantou University Medical College (Approval No. SUMC‐2021‐54) and was conducted in accordance with the 1964 Helsinki Declaration and its later amendments or comparable ethical standards. All participants provided written informed consent.

## Conflicts of Interest

The authors declare no conflicts of interest.

## Data Availability

Datasets used and/or analyzed during the current study are available from the corresponding author, Guanhua Fan, upon reasonable request.
